# Aortic Valve Sclerosis and Degenerative Valve Disease in a Nigerian Population: An Echocardiographic Study

**DOI:** 10.5334/gh.1455

**Published:** 2025-08-19

**Authors:** Olugbenga O. Abiodun, Ibrahim L. Salau, Tina Anya

**Affiliations:** 1 Department of Internal Medicine, Federal Medical Centre, Abuja, Nigeria; 2 Department of Internal Medicine, Baze University Abuja, Nigeria

**Keywords:** aortic sclerosis, calcific aortic valve disease, degenerative mitral valve disease, degenerative valve disease, non-rheumatic valve disease, Nigeria

## Abstract

**Background:**

The burden of degenerative valve disease is increasing globally because of population aging. However, data on this condition is scarce in Nigeria and across Africa.

**Objective:**

Our study evaluated the prevalence, pattern, and associations of aortic sclerosis and degenerative valve disease in a Nigerian population.

**Methods:**

Data of a total of 4030 patients were analyzed retrospectively from the Federal Medical Centre Abuja transthoracic echocardiographic registry from January 2020 to December 2024. Patients were 18 years and above. Aortic sclerosis, degenerative mitral valve disease, calcific aortic valve disease, and degenerative valve disease were defined in accordance with the report of the American Heart Association/American College of Cardiology guidelines.

**Results:**

The mean age of patients was 52 ± 15 years and 53.2% were female. The prevalence rates were 4.2% for aortic sclerosis, 0.2% for degenerative mitral valve disease, 1.2% for calcific aortic valve disease, and 1.3% for degenerative valve disease. Among patients aged 65 years and older, the prevalences rates were higher for aortic sclerosis (13.2%), degenerative mitral valve disease (1.0%), calcific aortic valve disease (3.4%), and degenerative valve disease (3.9%) [*P* < 0.001]. Only 1.7% and 0.6% of those <65 years had aortic sclerosis and calcific aortic valve disease, respectively. Patients aged 65 years and older, and hypertension had odds ratio (OR) of 7.99 and 3.98 for developing aortic sclerosis, respectively. For calcific aortic valve disease, the OR was higher for patients aged 65 years and older (OR: 4.25), hypertension (OR: 2.48), and left ventricular hypertrophy (OR: 5.35) [*P* < 0.001].

**Conclusion:**

In this Nigerian echocardiographic registry, age above 65 years and hypertension were associated with aortic sclerosis and calcific aortic valve disease.

## Introduction

Cardiovascular diseases (CVD) are the most common cause of morbidity and mortality globally. They are driven by cardiovascular (CV) risk factors, population growth, and aging (1). With increasing age, degenerative valve disease (DVD), namely calcific aortic valve disease (CAVD), and degenerative mitral valve disease (DMVD) are the two most commonly seen non-rheumatic valve disease (NRVD) (1,2,3). In 2017, CAVD and DMVD accounted for 102,700 and 35,700 deaths, and 12.6 million and 18.1 million prevalent cases, respectively (1). The prevalence of NRVD is rising globally, particularly in countries with high sociodemographic index (SDI), due to an aging population. The prevalence is <2% until the age of 64 years, but increases to 12%–13% after 75 years of age (1,2,3,4). Additionally, mortality from DMVD is higher among patients over 65 years, peaking after the age of 85 globally (2).

Aortic sclerosis is the precursor of CAVD, and according to the Cardiovascular Health Study, 9% of patients aged 65 years or older will progress to aortic stenosis within 5 years (5). Similarly, the incident rate of aortic sclerosis was reported to increase by a mean of 1.7% per year among 5880 participants aged 45 to 84 years in the Multi-Ethnic Study of Atherosclerosis (MESA) (6).

Risk factors that may predispose to DVD include increasing age, increased body mass index, current smoking, higher levels of low-density lipoprotein cholesterol, male sex, hypertension, and diabetes (7,8). While DVD share similar risk factors with atherosclerotic heart disease, statins have not demonstrated any preventive benefit (7,8). Further research buoyed by the risk factor paradox has led to the implication of osteogenic processes as key mechanisms in the development of DVD (4).

In Africa, there is limited data on aortic sclerosis and DVD, as the focus continues to be on rheumatic heart disease (RHD) which is estimated to account for 15.6–19.6 million cases and 233,364–294,398 deaths each year (3,9,10). Consequently, there is a significant knowledge gap regarding DVD. This study aims to address this gap by providing echocardiographic data on the prevalence, patterns, and associations of DVD in a Nigerian population. The findings will help determine the extent of the burden of DVD and equip clinicians and policy makers with important information to plan and make provisions for preventive and curative services.

## Materials and Methods

Data from a total of 4030 patients from the Federal Medical Centre Abuja (FMCA) transthoracic echocardiographic registry from January 2020 to December 2024 were retrospectively analyzed. Data on age, sex, anthropometry (height, weight, and body surface area), indication for echocardiography, and echocardiographic parameters were retrieved from the registry. Duplicate entries were identified by patients’ names and hospital number and only the first study was included in the registry. The FMCA holds two echocardiographic sessions per week carried out by three cardiologists and their residents. Measurements taken followed recommendations of the American Society of Echocardiography (ASE) and the European Association of Cardiovascular Imaging (EACVI) (11). Echocardiography was performed by three cardiologists and their residents using General Electric Model Vivid E9, Horten, Norway with M5S probe or General Electric, Model S6, Horten, Norway with M4S probe. Data was stored in an Excel database and studies were reported after each session by the cardiologists and their residents. Echocardiographic parameters retrieved from the database for this study included interventricular septal thickness in diastole (IVSTD), left ventricular posterior wall thickness in diastole (PWTD), interventricular septal dimension in systole (IVSS), left ventricular internal dimension in diastole (LVIDD), relative wall thickness (RWT), left ventricular mass index (LVMI), linear ejection fraction, fractional shortening, mitral peak E wave velocity (E), mitral peak A velocity (A), mitral E/A ratio, and tissue doppler-derived mitral annular e′ velocity (e′), E/e′ ratio, report summaries, and conclusions. The indications for the echocardiographic studies were also retrieved. Left ventricular mass index was calculated using the linear cube formula of the American College of Cardiology: 0.8 (1.04 ([LVIDD + PWTD + IVSTD]^3^ – [LVIDD]^3^))+ 0.6 g and indexing with body surface area (BSA) (11). Relative wall thickness (RWT) was calculated with the formula: 2 × PWTD/LVIDD. Ethical approval with number FMCABJ/HREC/2024/162 was obtained from the hospital’s ethics research committee.

## Definitions

– Obesity was defined as body mass index of 30 kg/m^2^ and above (12).
– Left ventricular hypertrophy was defined as LVMI of more than 95 g/m^2^ in females and 115 g/m^2^ in males (11,13).
– Hypertension, stroke, DM, and IHD were diagnosed by hospital physicians and the cardiologists according to guideline recommendations (14,15,16,17).
– Aortic sclerosis was defined as thickening and/or calcification of the aortic valve leaflets with a transvalvular velocity of <2 m/s (18).
– Calcific aortic valve disease was defined as calcification of the aortic valve leaflets or annulus with mild to severe stenosis (transvalvular velocity of >2.5 m/s) and or regurgitation in the absence of commissural fusion and thickening or calcification along the edges of the valve cusps (1,18).
– Degenerative mitral valve disease was defined as myxomatous degeneration of the mitral valve with moderate or severe regurgitation in the absence of commissural fusion and involvement of other parts of the mitral valve apparatus (1,18,19).
– Degenerative valve disease was defined as the presence of any of DMVD and CAVD (1,18).

## Data Analysis

Continuous variables were expressed as means ± standard deviation, while categorical variables were expressed as numbers and percentages. The association of variables of interest in those aged <65 years and those aged 65 years and older was tested by χ^2^ for categorical variables and independent *t*-test for continuous variables. Fisher’s exact test was used for categorical variables with an expected cell size of <5. Multivariable logistic regression with model-fitting statistics was used to draw the association between statistically significant associated variables from univariable models and the outcomes of interest namely aortic sclerosis and CAVD. *P*-value <0.05 were taken as statistically significant. Data was analyzed using the Statistical Package for Social Sciences (SPSS) version 26 software for Windows.

## Results

Table 1 shows the baseline characteristics of patients categorized by age. The mean age of patients was 52 ± 15 years with a female preponderance of 53.2% compared to 46.8% for males. Among patients aged 65 years and older, there were significantly higher rates of hypertension, stroke, DM, IHD, LVH, aortic sclerosis, DMVD, CAVD, and DVD (*P* < 0.05). However, patients younger than 65 years showed higher rates of obesity than those aged 65 years and older (*P* < 0.05). Among all the patients, the prevalence of aortic sclerosis was 4.2%. However, it was significantly higher at 13.2% in those aged 65 years and older, compared to 1.7% in those younger than 65 years (*P* < 0.001). The prevalence of DMVD was 0.2%, but this rose to 1.0% in the older group, while there were no cases reported in those younger than 65 years (*P* < 0.001). For CAVD, the overall prevalence was 1.2%, with rates significantly higher at 3.4% in individuals aged 65 years and older compared to 0.6% in those younger than 65 years (*P* < 0.001). Among CAVD cases, half were mild (0.6%) while the remaining half were equally distributed between moderate (0.3%) and severe CAVD (0.3%) cases. The overall rate of DVD was 1.3%, with 3.9% of cases in patients aged 65 years and older compared to 0.9% of cases in those younger than 65 years (*P* < 0.001).

**Table 1:** Baseline characteristics of patients.


VARIABLES	TOTAL *N* = 4030	<65 YEARS *N* = 3148 (78.1%)	≥65 YEARS *N* = 882 (21.9%)	*P*-VALUE

**Sociodemographic**				

Age (years)	52 ± 15	47 ± 11	73 ± 7	<0.001

Sex				0.833

Male	1886 (46.8%)	1476 (46.9%)	410 (46.5%)	

Female	2144 (53.2%)	1672 (53.1%)	472 (53.5%)	

**Clinical**				

Obesity	1649 (45.4%)	1361 (46.4%)	288 (41.3%)	0.003

Body mass index (kg/m^2^)	29.8 ± 6.1	30.0 ± 6.1	29.2 ± 6.0	0.153

Hypertension	2167 (53.8%)	1620 (51.5%)	547 (62.0%)	<0.001

Stroke	60 (1.5%)	39 (1.2%)	21 (2.4%)	0.013

Diabetes mellitus	143 (3.5%)	97 (3.1%)	46 (5.2%)	0.002

Ischemic heart disease	58 (1.4%)	27 (0.9%)	31 (3.5%)	<0.001

**Echocardiographic**				

Left atrial diameter (mm)	35.7 ± 6.7	35.5 ± 6.6	36.2 ± 7.0	0.011

Aortic root diameter (mm)	29.2 ± 4.9	29.0 ± 5.0	29.8 ± 4.6	<0.001

IVSTD (mm)	12.5 ± 3.0	12.3 ± 3.0	13.2 ± 3.2	<0.001

PWTD (mm)	10.6 ± 2.5	10.5 ± 2.3	10.9 ± 2.4	<0.001

LVIDD (mm)	48.0 ± 8.4	48.4 ± 8.1	46.8 ± 9.1	<0.001

LVIDS (mm)	30.3 ± 9.3	30.4 ± 9.1	29.8 ± 9.9	0.075

RWT	0.46 ± 0.1	0.45 ± 0.1	0.49 ± 0.2	<0.001

LVMI (g/m^2^)	112.5 ± 41.6	110.7 ± 40.7	120.1 ± 44.3	<0.001

LVH	1830 (50.5%)	1421 (48.5%)	409 (58.8%)	<0.001

Ejection fraction (%)	66.0 ± 14	67.0 ± 13.0	65.0 ± 15.0	0.016

Fractional shortening (%)	38.0 ± 10.0	38.0 ± 10.0	37.0 ± 11.0	0.057

E velocity (m/s)	0.82 ± 3.3	0.84 ± 3.6	0.77 ± 2.1	0.575

A velocity (m/s)	1.34 ± 23.7	1.41 ± 26.5	1.10 ± 6.1	0.553

E/A	1.26 ± 2.2	1.28 ± 1.6	1.21 ± 3.7	0.602

E′ velocity (m/s)	0.15 ± 2.4	0.17 ± 2.8	0.09 ± 0.2	0.120

E/E′	9.4 ± 15.6	8.96 ± 14.4	10.94 ± 19.0	0.002

Aortic sclerosis	169 (4.2%)	53 (1.7%)	116 (13.2%)	<0.001

Degenerative mitral valve disease	10 (0.2%)	1 (0.0%)	9 (1.0%)	<0.001*

Calcific aortic valve disease	48 (1.2%)	18 (0.6%)	30 (3.4%)	<0.001

Mild	25 (0.6%)	9 (0.3%)	16 (1.8%)	

Moderate	12 (0.3%)	3 (0.1%)	9 (1.0%)	

Severe	11 (0.3%)	6 (0.2%)	5 (0.6%)	

DVD	53 (1.3%)	19 (0.6%)	34 (3.9%)	<0.001


IVSTD, interventricular septal thickness in diastole; PWTD, left ventricular posterior wall thickness in diastole; LVIDD, left ventricular internal dimension in diastole; LVIDD, left ventricular internal dimension in systole; RWT, relative wall thickness; LVMI, left ventricular mass index; LVH, left ventricular hypertrophy; DVD, degenerative valve disease. *Fishers exact test.

Patients aged 65 years and older showed significantly greater dimensions of LA, AO, IVSTD, PWTD, RWT, LVMI, ejection fraction, and E/E′ but significantly lower LVIDD (*P* < 0.05). There was no significant difference between the two age groups for BMI, LVIDS, fractional shortening, E velocity, A velocity, E/A ratio, and E′ velocity (*P* > 0.05).

Table 2 shows the multivariable logistic regression analysis of co-variates and aortic sclerosis, which indicates that individuals aged 65 years and older (OR: 7.99) and hypertension (OR: 3.98) significantly increase the odds of aortic sclerosis (*P* < 0.001).

**Table 2:** Multivariable logistic regression of co-variates and aortic sclerosis.


AORTIC SCLEROSIS		

VARIABLES	ODDS RATIO (CONFIDENCE INTERVAL)	*P*-VALUE

Age ≥65 years	7.99 (5.51–11.59)	<0.001

Sex	0.89 (0.62–1.29)	0.540

Obesity	0.78 (0.54–1.13)	0.185

Hypertension	3.98 (2.44–6.49)	<0.001

Diabetes	1.87 (0.93–3.77)	0.078

Stroke	2.26 (0.61–8.35)	0.221

Ischemic heart disease	1.49 (0.41–5.39)	0.540

Left ventricular hypertrophy	1.61 (1.10–2.34)	0.672


Model fit of *P* < 0.001, Cox & Snell *R*^2^ of 0.058, Nagelkerke *R*^2^ of 0.207, Homer and Lemeshow test of 0.262.

Table 3 shows the multivariable logistic regression analysis of co-variates and CAVD. Age 65 years and older (OR: 4.25), hypertension (OR: 2.48), and LVH OR: (5.35) were found to significantly increase the odds of CAVD (*P* < 0.001).

**Table 3:** Multivariable logistic regression of co-variates and calcific aortic valve disease.


CALCIFIC AORTIC VALVE DISEASE		

VARIABLES	ODDS RATIO (CONFIDENCE INTERVAL)	*P*-VALUE

Age ≥65 years	4.25 (2.17–8.30)	<0.001

Sex	0.82 (0.42–1.61)	0.564

Obesity	0.53 (0.26–1.08)	0.079

Hypertension	2.48 (1.10–5.63)	0.029

Diabetes	1.34 (0.31–5.89)	0.698

Stroke	2.66 (0.33–21.21)	0.357

Ischemic heart disease	1.66 (0.21–13.43)	0.635

Left ventricular hypertrophy	5.35 (2.07–13.85)	0.001


Model fit of *P* < 0.001, Cox & Snell *R*^2^ of 0.015, Nagelkerke *R*^2^ of 0.136, Homer and Lemeshow test of 0.060.

## Discussion

The prevalence of aortic sclerosis, DMVD, CAVD, and DVD in this Nigerian population were 4.2%, 0.2%, 1.2%, and 1.3%, respectively. Our study also revealed that age 65 years and older, and hypertension were associated with aortic sclerosis and CAVD in this population. To the best of our knowledge, this is the first African study to report a focused evaluation of aortic sclerosis, CAVD, DMVD, and DVD. While rheumatic heart disease remains the focus of valvular heart disease in Africa, the ongoing steady aging transition due to population growth and aging suggests that DVD may increasingly contribute to more heart failure and mortality in the coming years.

In this study, the prevalence rate of aortic sclerosis in our general echocardiographic population as well as individuals aged 65 years and above falls within the prevalence range reported in a systematic review and meta-analysis by Coffey et al., which examined 22 studies and found rates between 2.33% and 51.7% (20). The wide prevalence range may reflect the different populations sampled, and their age distributions and the modality used in diagnosing aortic sclerosis. In the Hypertension Genetic Epidemiology Network Study with similar mean age as our study, Agno et al. reported a prevalence rate of 9.4% for aortic sclerosis among 1624 hypertensive participants (21). It is important to note that aortic sclerosis is not merely an innocent precursor to CAVD, but a marker of coronary atherosclerosis, increased CV risk and mortality (20,22,23,24). Consequently, patients with aortic sclerosis should be regarded as high-risk patients and should be risk-stratified, offered aggressive treatment for modifiable CV risk factors and closely followed up to reduce CV morbidity and mortality.

In the Cardiovascular Health Study (CHS), 5888 participants with a mean age of 72.5 years were followed up for 5 years. The prevalence of aortic sclerosis was 29%, which is significantly higher than the 13.2% observed in our study among individuals aged 65 years and older with similar mean age (25). Unlike our study, the CHS prospectively followed up participants aged 65 years and older, which may explain the differences in the rates observed. Additionally, the CHS reported that 9% of patients with aortic sclerosis progressed to CAVD over the same 5-year period. Prospective studies are needed to examine the pattern of progression from aortic sclerosis to CAVD among Nigerian and African patients.

In our study, the overall prevalence of CAVD was 1.2%, increasing to 3.4% in patients aged 65 years and older. This is similar to the 3% observed in a cross-section of patients aged 65 years and above in a Mediterranean region (26) but lower than the rate of 5% reported in the US population at the same age (27). Life expectancy at birth in Nigeria increased by 9.34 years, from 54.1 years in 2000 to 63.4 years in 2021. If this trend continues, the prevalence and burden of CAVD are likely to increase in the coming years (28). Given the limited access to cardiac surgical services principally due to inadequate healthcare funding in Nigeria, public health, and clinical efforts should be geared toward preventing and retarding aortic sclerosis and DVD.

Our echocardiographic registry study showed that individuals aged 65 years and older and those with a history of hypertension are at increased risk for aortic sclerosis and CAVD. This finding is consistent with studies by Ferreira-González et al. and the CHS of Novaro et al. (25,26). In a random sample of 1068 individuals aged 65 years and older in Spain reported by Ferreira-González et al., age increased the odds of mild-to-moderate and moderate-to-severe aortic sclerosis by 1.56 and 2.03, respectively. Similarly, Navaro et al. reported an odd of 1.13 risk for incident aortic stenosis with increasing age in the CHS study (25). Our findings of a significant increase in the odds of aortic sclerosis by 7.99 and 3.98 and CAVD by 4.25 and 2.48 in individuals aged 65 years and above and hypertension suggests that elderly hypertensives in our population could benefit from echocardiographic screening to identify aortic sclerosis and DVD. Early identification, followed by appropriate monitoring of aortic sclerosis and CAVD, is essential for the timely diagnosis of HF and reduction of the overall burden of CV disease in this age group.

Our study demonstrates that LVH is associated with the risk of CAVD. This finding aligns with the MESA study conducted by Elmariah et al., which found that LVH is a strong predictor of incident valve calcification (29). LVH has been linked to aortic calcification even in the absence of stenosis, suggesting that alternate mechanisms involving inflammatory and neurohormonal factors may contribute to calcification due to LVH (29). Therefore, early detection of LVH and its regression through appropriate medications may be essential for preventing the development of valve calcification and CAVD.

There are several limitations to consider when interpreting the results of this study. First, our study is a single center study and may not be generalizable to the entire Nigerian population. However, there is limited literature on aortic sclerosis and DVD in African and Nigerian populations, and this study will encourage further research in this overlooked area compared to rheumatic valve disease. Second, our study utilized transthoracic echocardiography to assess degenerative valve changes. While cardiac CT may provide additional information on the extent of valvular calcification, echocardiography remains essential for confirming the diagnosis and severity of valvular diseases, including calcification (30). Third, our research was based on an echocardiographic registry, which limited data on predictors of these valve lesions. The evaluation of hypertension was the primary reason for requesting echocardiography in our registry. To address potential bias in interpreting the association between NRVD and hypertension, we conducted a multivariable logistic regression analysis and reported adjusted estimates for hypertension. Lastly, we focused on only two types of NRVD namely CAVD and DMVD. Other non-rheumatic aortic and mitral valvular lesions were scarce in our echocardiography registry, and lesions affecting the tricuspid and pulmonary valves were rare as well.

## Conclusion

Our study shows that the prevalence of aortic sclerosis and CAVD in this Nigerian cohort is similar to findings from other regions with longer life expectancies. The main associated factors of these degenerative valve lesions are increased age and the presence of hypertension, both of which have been widely documented in the literature. As life expectancy continues to rise, the burden of DVD is expected to increase, which will significantly strain healthcare resources that are already insufficient for younger populations. Further research is therefore essential to identify effective therapeutic options for preventing DVD. In the meantime, early diagnosis and proper management of hypertension and LVH, as well as monitoring and treatment of patients with aortic sclerosis and CAVD where indicated are crucial for reducing CV morbidity and mortality.

## Data Accessibility Statement

The authors declare the availability of the dataset for this study upon reasonable request.
